# Facial features associated with fear and happiness attract gaze during brief exposure without enhancing emotion recognition

**DOI:** 10.1038/s41598-025-12327-6

**Published:** 2025-08-19

**Authors:** Yu-Fang Yang, Matthias Gamer

**Affiliations:** 1 Department of Psychology, University of Würzburg, Würzburg, Germany; 2 Division of Experimental Psychology and Neuropsychology, Department of Education and Psychology, Freie Universität Berlin, Berlin, Germany

**Keywords:** Facial expression, Emotion, Facial features, Eye-tracking, Peripheral vision, Psychology, Human behaviour

## Abstract

Facial features transmit emotions but their effect on visual orienting and explicit emotion recognition is debated. Here we examined whether fixating on diagnostic features of emotional expressions—such as eye region for fear and the mouth for happiness—affects saccadic targeting and improves recognition accuracy. Across two pre-registered experiments, participants viewed fearful, happy, and neutral faces for short intervals (50 or 150 ms) while the initial fixation location was manipulated. Although such brief stimulation does not allow for visual exploration, the faces still elicited reflexive saccades that occurred after stimulus offset. These saccades were modulated by the emotional expressions indicating a consistent preferential saccadic orienting towards diagnostic features, even with limited exposure. As this effect disappeared for inverted faces, it can be attributed to an extrafoveal processing of facial features instead of an attentional orienting towards physically salient image regions. Participants’ recognition accuracy was unaffected by the foveated facial feature, but this observation might also be due to ceiling effects in performance. Collectively, these findings contribute to understanding the attentional mechanisms of feature-based processing in the perception of emotional facial expressions.

## Introduction

During everyday life, we often have limited time to process an individual’s facial expression as one approaches. However, accurate decoding of facially expressed information is critical to respond appropriately and promptly in social contexts. Introspection suggests that we are usually capable of recognising an emotional expression during a quick glance at a face. Studies measuring eye movements or event-related brain potentials (ERPs) confirmed that facial expression processing and identification are so rapid^1–3^ that the involvement of a pre-attentive mechanism seems likely. Interestingly, the information that is needed to accurately recognise emotional expressions is distributed across the face and differs between emotions^4–6^. Several studies have shown that human observers tend to prioritise diagnostic facial features, such as the eyes for fear or the mouth for happiness, during emotion perception^3,7–10^. However, it remains unclear to what extent this preferential processing actually improves the recognition of emotional facial expressions. Some studies have reported faster emotion recognition when participants fixated emotion-diagnostic features using foveal vision (e.g.,^11^), whereas others found improved classification accuracy only for specific emotions, such as anger or surprise (e.g.,^4^). This discrepancy might be related to crucial differences in the experimental design, for example concerning the presentation time or the selection of foveated features.

### Impact of presentation time and visual exploration on facial expression classification

Human observers can identify individuals within one or two fixations^12^. Similarly, they can discriminate between emotional facial expressions with remarkable speed (e.g.,^13^), often achieving accurate recognition within less than 200 ms, as also substantiated by electroencephalogram (EEG) measures^15^. Using a forced-choice emotion detection paradigm with a backward mask, Kirouac & Dore^16^ were the first to demonstrate that participants can accurately recognize the basic six emotional facial expressions (i.e., happiness, sadness, anger, fear, disgust, surprise) under conditions of limited exposure times ranging from 20 to 50 ms. Concerning accuracy rates, performance in detecting emotional facial expression is enhanced with longer presentation times, as more information can be collected^3,16^. Using a similar paradigm, Esteves and Öhman^17^ presented neutral, angry, or happy faces for a duration of 20 to 300 ms, followed by an upright neutral-face mask. Their study revealed that emotion discrimination performance improved as presentation time increased and that an exposure of approximately 100 ms was necessary for recognizing facial expressions with high accuracy. On the contrary, presentation durations shorter than 50 ms were associated with relatively poor recognition performance. A consistent finding across the aforementioned studies is the happy-superiority effect: observers usually recognise happy faces faster and more accurately compared to other facial expressions within exposure times as short as 50 ms^3^. Recognition rates of fear or surprise, by contrast, are typically lower than for other emotional expressions regardless of whether masking is applied or not^3,18^.

Accurate emotion recognition under brief exposure appears to be facilitated by holistic face processing that allows integrating distributed information during the early stages of visual analysis^15,19^. However, also specific facial features seem to be important for recognizing emotional expressions, as shown in several behavioural studies using the so-called ‘bubbles technique’—a method that systematically limits facial information by revealing selected regions and spatial frequency bands through Gaussian apertures^6,20^. Although this approach does not entirely disrupt holistic processing^22^, it has been demonstrated that diagnostic features for recognizing emotional expressions are differentially distributed across the face. For example, the eyes are more diagnostic for recognising fear, whereas the mouth area seems more relevant to accurately categorize happy facial expressions^6,10^. Assuming that our visual system might be sensitive to the distribution of such features in the visual periphery, it seems interesting to examine whether participants show increased attentional orienting towards these locations.

### Modulation of first saccades by diagnostic features

Previous findings generally support the idea that expressive features modulate the visual exploration of facial expressions. When being able to explore faces comprehensively (i.e., for several seconds), observers typically show a triangular exploration pattern with most fixations landing on the eye, mouth and nose region^21^. However, this pattern is not constant, but modulated by emotional expression. For example, the eye region is typically explored more extensively for fearful, angry, and sad expressions, while the mouth region receives relatively more attention for happy faces^8,10^. To examine more reflexive aspects of such attentional effects, several studies restricted the viewing time of faces to less than 200 ms and manipulated the initial fixation on the stimulus by unpredictably shifting facial expressions relative to the previously shown fixation cross. Importantly, under such conditions, it is not possible to visually explore the stimulus, as it already disappeared from the screen before the first saccade reaches a new fixation target. However, these studies still showed a substantial number of saccades that were obviously triggered by the stimulus, occurred after stimulus offset, and were modulated by the initial fixation and the depicted emotional expression. In detail, observers were shown to generally perform more upward saccades toward the eye region than downward saccades towards the mouth^10^, pointing to a special role of the eye region during face processing^23^. Interestingly, these trends become more apparent when observers process fearful, angry, or neutral faces compared to happy expressions, suggesting that observers reflexively direct their attention toward diagnostic features^9,33^).

Although these effects were replicated in several studies, two important shortcomings should be mentioned. First, it seems very difficult to control for the influence of physical saliency in such studies. Thus, an increased orienting towards the eye region could simply be due to a general sensitivity for brighter image regions such as the eyes^24^. This might also partly explain emotion-specific effects since the eye region is usually brighter for fearful faces and the mouth region for happy expressions. Although previous studies partly addressed this potential confound by controlling for differences in physical saliency^10^, this issue might still explain some of the aforementioned findings. A relatively easy way to control for such effects seems to be the use of inverted faces. They have a similar distribution of physical saliency, but impair the processing of facial configurations, resulting in substantially degraded emotion recognition^19,25^. To the best of our knowledge, it has not yet been examined to what degree face inversion affects the attentional orienting towards diagnostic features. Second, contrasting previous reports, two recent studies found that reflexive initial saccades did not preferentially target expression-specific diagnostic features regardless of the depicted emotion^4,31^. Although the experimental design and the task were roughly similar to previous studies, some differences are noticeable that might explain the divergent results: 1) The studies by Atkinson & Smithson^4^ and Duran & Atkinson^31^partly included a different set of emotional expressions than previous studies which mostly focused on fearful, angry, happy, and neutral faces (e.g.,^33^) since these expressions differ most clearly in the distribution of diagnostic features^6^. 2) Whereas individual faces were only shown once or twice in previous studies, each face was presented four times in the experiments by Atkinson and Smithson^4^ and Duran and Atkinson^31^. While repeated exposure can raise the possibility of memory effects, supplementary analyses in Atkinson and Smithson^4^ found no interaction between image serial position and the critical effects of fixation location and emotional expression. Thus, repetition alone is unlikely to explain the divergent results but should be examined in more detail in future studies by explicitly manipulating the number of repetitions. 3) Faces were cropped in previous studies to exclude features that are not diagnostic for emotional expressions (e.g., hair or ears) whereas this was not accomplished in the work by Atkinson and colleagues. 4) Although all the respective studies used a brief presentation of emotional expressions, Atkinson & Smithson^4^ and Duran & Atkinson^31^used a substantially shorter presentation time—approximately 80 ms compared to 150 ms (e.g.,^9,10^). 5) Previous studies typically restricted initial fixations (i.e., foveated features) to two locations (centre of the eye and mouth region) whereas a more fine-grained pattern with 6 initial fixations (e.g., including cheeks) was used by the studies of Atkinson and colleagues.

### The present study

To clarify the influence of foveated features on visual exploration and emotion recognition, we tested the influence of two critical manipulations that differed between previous studies in two pre-registered experiments. In Experiment 1, we systematically varied the presentation time (50 vs. 150 ms) and additionally implemented a condition with inverted faces to control for effects of low-level features (e.g., brightness). In Experiment 2, we investigated to what extent the number of possible initial fixations (two vs. six locations) influenced reflexive saccades and emotion recognition.

For Experiment 1, we expected a preferential visual orienting towards diagnostic features. Thus, we hypothesised to observe that 1) first saccades after stimulus onset would be sensitive to the emotional expression with relatively more saccades towards the eye region occurring for fearful and neutral faces and relatively more saccades towards the mouth region appearing for happy faces. However, since this effect was not observed in two recent studies with very short presentation times^4,31^, 2) we expected the effect to be weaker for 50 ms than for 150 ms. Inverting faces impairs the processing of facial configurations and hinders an accurate detection of the location of specific features in the visual periphery while preserving the distribution of physical saliency. Hence, 3) we assumed that the effect described in hypothesis 1 is less pronounced for inverted than for upright faces, which would also rule out that previously observed effects were solely due to differences in physical saliency. Furthermore, we tested the influence of the experimental manipulations on emotion recognition accuracy but since previous studies reported rather inconsistent results concerning the influence of foveated features on recognition performance, we restricted our hypotheses to rather general effects and 4) assumed the accuracy of emotion classification to be higher for happy than for neutral and fearful faces and for upright as compared to inverted faces.

For Experiment 2, we predicted to replicate a general preference of showing more saccades toward the eye region than towards the mouth, with the pattern again being modulated by the emotional expression similar to Experiment 1. Since this effect did not emerge in two recent studies^4,31^, we expected it to be weaker in the condition with six possible initial fixation locations compared to the two locations previously used. Regarding the behavioural data, we again assumed a more accurate classification of happy expressions and exploratively analysed the effect of the other experimental manipulations on classification accuracy.

## Experiment 1

### Methods

#### Transparency and openness

For this and the subsequent experiment, we describe how sample size was determined, we report all data exclusions, all manipulations, and all measures in the study, and we follow the APA Style Journal Article Reporting Standard^26^. The experiments were programmed in Presentation (Neurobehavioral Systems, Berkeley, CA, USA). Data analysis was conducted using the R environment for statistical computing and plotting (R Core Team^27^, version 4.3.2). The tidyverse^28^, afex^29^ and lsmeans^30^ packages were used for data processing and statistical analyses (ANOVA and post-hoc analysis). Trial-wise data and analysis scripts are available on the OSF (https://osf.io/g2sk9/). Experimental designs, sample sizes and analysis plans of Experiment 1 (https://aspredicted.org/ZZT_CGV) and Experiment 2 (https://aspredicted.org/DWD_EGT) were preregistered before data acquisition. All methods were carried out in accordance with relevant guidelines and regulations, including the Declaration of Helsinki, and the study was approved by the Ethics Committee of the Department of Psychology at the Julius-Maximilians-University of Würzburg (GZEK 2020–39).

#### Participants

In contrast to previous studies that frequently relied on sample sizes around 30 participants^4,10,31^. we pre-registered a total sample size of 60 participants for the current experiment. This was based on a simulation^32^ using estimates of *M*, *SD*, and the correlation between factor levels from our previous work^33^, which indicated a power of ~ 0.80 for detecting interaction effects between the newly introduced experimental manipulations of face inversion and presentation time with the two-way interaction of emotional expression and initial fixation (see supplementary material for further details).

A total of 64 participants were recruited. However, due to less than 70% valid trials (see below), four participants had to be eliminated from the data analysis, resulting in a final sample of 60 participants (age: *M* = 26.2 years, *SD* = 8.4 years). The sample included 19 males, 40 females and one person who identified as diverse. All participants gave written informed consent to participate in the study, including consent for the publication of non-identifying data and information in an online open-access publication, and were informed about their rights regarding data protection. All had normal or corrected normal vision using soft contact lenses and most of them were students (80%). Participants were mainly recruited through an online database for psychological studies of the University of Würzburg, and either received course credits or a monetary compensation of 10 €.

#### Stimuli and design

The stimulus set used in this experiment was the same as in Scheller et al.^10^. It included stimuli from different databases (The Karolinska directed emotional faces, KDEF; Pictures of facial affect; Nim-Stim; FACES database) that unequivocally depicted the respective emotional expression as verified by validation studies^34,35^. The faces were slightly rotated to keep both pupils constantly on the same imaginary horizontal line. Afterwards, they converted to greyscale and cropped using an elliptical mask to remove non-expressive information (i.e., hair and ears). Finally, the luminance of the pictures was normalized across all images. The final set included 126 fearful, 144 happy, and 139 neutral facial expressions. From this pool, 96 images per emotional expression (48 women, 48 men) were randomly selected for each participant. An additional set of eight images for each emotional expression was randomly selected for a short training block to familiarize participants with the task.

The stimuli were randomly associated to the experimental conditions that were defined by the factors *emotional expression* (fearful, happy, neutral), *presentation time* (50, 150 ms), *orientation* (upright, inverted), and *initial fixation* (eyes, mouth) while ensuring for an equal number of male and female faces in each condition. This resulted in 12 trials for each factor combination and 288 trials in total. Importantly, each picture only appeared on one trial within each participant and was never repeated. A similar assignment was conducted for the training block that included a total of 24 trials (one trial for each factor combination). For the main experiment, the trials were assigned to three blocks with 96 stimuli each to allow for short breaks.

#### Apparatus

The study was programmed using the software Presentation (Neurobehavioral Systems, Berkeley, CA, USA) and stimuli were displayed on an LG 24MB65PY-B screen (51.69 × 32.31 cm) with a resolution of 1920 × 1200 pixels and a refresh rate of 60 Hz. The experiment was carried out in a soundproof booth with dim light, with eye movements of the right eye tracked at a sampling rate of 500 Hz using an EyeLink 1000 Plus system (SR Research, Ottawa, ON, Canada) in the remote setup. This allowed for head free eye tracking with a distance of approximately 60 to 65 cm between the eyes of the participants and the centre of the presentation screen. Under these conditions, the visual angle of the stimuli amounted to approximately 12.12° × 19.45°.

#### Procedure

This experiment was carried out during the COVID-19 pandemic and therefore included several safety measures to reduce unnecessary risks to the participants. Before the experiment started, participants were given a questionnaire concerning potential symptoms or risks of a COVID-19 infection (e.g., contact to infected individuals during the previous two weeks) and they were only allowed to participate when no risks could be identified, and participants reported to be healthy. Afterwards, they were informed about the experimental procedures and the general purpose of the study. The experiment began with the training block consisting of 24 trials. Following this, the experiment started, and participants completed three blocks of 96 trials each.

**Fig. 1:**
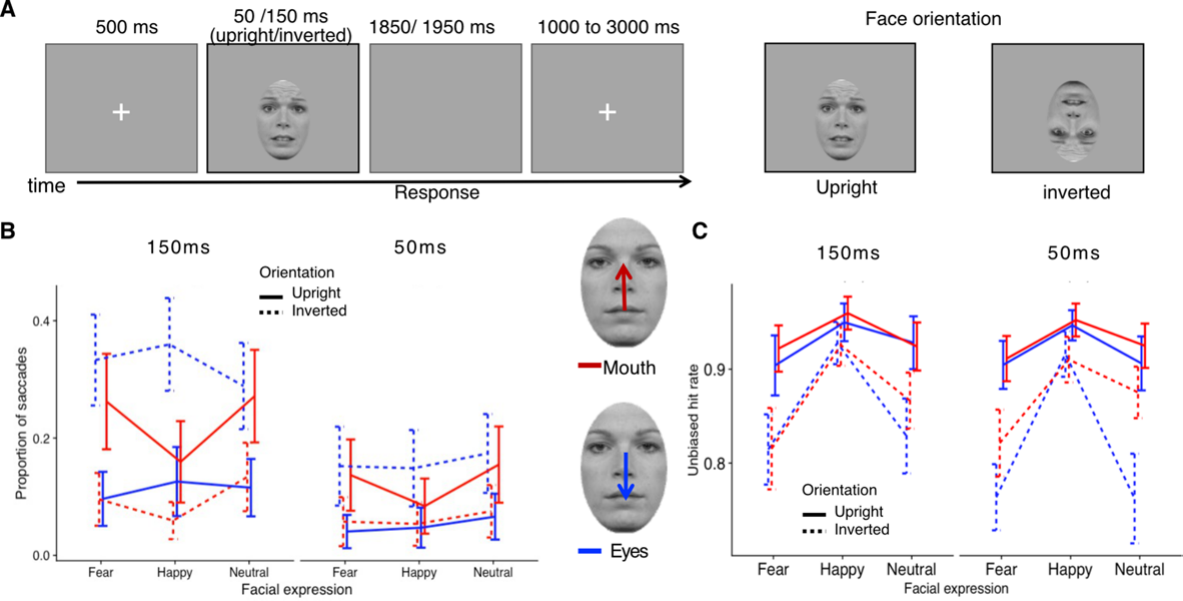
Depiction of the experimental manipulations and the results of Experiment 1. (**A**) Schematic illustration of the trial structure and the manipulation of the face orientation. (**B**) Proportion of saccades after stimulus onset as a function of face orientation, emotional expression, and initial fixation for 150 and 50 ms presentation times. (**C**) Unbiased hit rates in the emotion recognition task as a function of face orientation, emotional expression, and initial fixation for both presentation times. Error bars indicate 95% confidence intervals.

A 9-point calibration and validation procedure of the eye-tracker was performed at the beginning of each block. Each trial (see Fig. 1A) started with a white fixation cross in the centre of a grey background for 500 ms. Following this, the face (upright or inverted) appeared for 50 or 150 ms with participants either fixating the centre of the eye or mouth region. This manipulation was realized by vertically shifting the faces relative to the previously shown fixation cross. After face offset, a grey background was shown for either 1950 or 1850 ms depending on the face presentation time before the fixation cross appeared again for a variable duration of 1000 to 3000 ms (uniform distribution). Participants were instructed to classify the emotional expression as fast and accurately as possible using the keys one, two and three of the number pad of a standard computer keyboard. Assignment of keys to emotional expressions was fixed and learned during the initial training block. Participants were furthermore instructed to fixate the fixation cross whenever it was visible on the screen and to avoid excessive blinking. Within each block, trials were presented in a random order, with the restriction that the same emotional expression never occurred in more than three successive trials.

### Data processing and analysis

#### Eye-tracking data

In a first step, the software DataViewer (SR Research, Ottawa, ON, Canada) was used to extract saccades and fixations from the eye-tracking data using default parameters. A saccade was defined as a change in eye position with a velocity of more than 30°/s or an acceleration of at least 8,000°/s^2^. Fixations were defined as periods of relatively stable gaze between saccades.

To ensure that participants were fixating at the fixation cross at trial start, we determined the gaze location in a baseline period of -300 to 0 ms before stimulus onset. We then applied a recursive outlier detection approach on these baseline values separately for x- and y-coordinates of each experimental block (for a similar approach see^36,37^. Although some previous studies (e.g.,^4^) used fixation-contingent stimulus presentation to enforce compliance, we opted for a fixed stimulus onset with post-hoc validation. This approach ensured uniform stimulus timing across trials—particularly critical for short presentation durations—while still enforcing strict fixation accuracy. To assess baseline validity, we temporarily removed the minimum and maximum of the respective distribution of x- and y-coordinates and calculated the mean and standard deviation of the remaining values. Minimum and/or maximum values were defined as outliers and permanently removed if they deviated more than 3 *SD*s from the mean. This procedure was repeated until no more values were defined as outlier. Trials with baseline outliers or blinks between −300 and 150 ms relative to stimulus onset were excluded from all analyses. According to our preregistered criterion, four participants of the original sample were excluded because less than 70% of trials remained for the analyses (see participants section above). In the final sample, 9.1% of trials were excluded on average (*SD* = 5.4%).

**Fig. 2:**
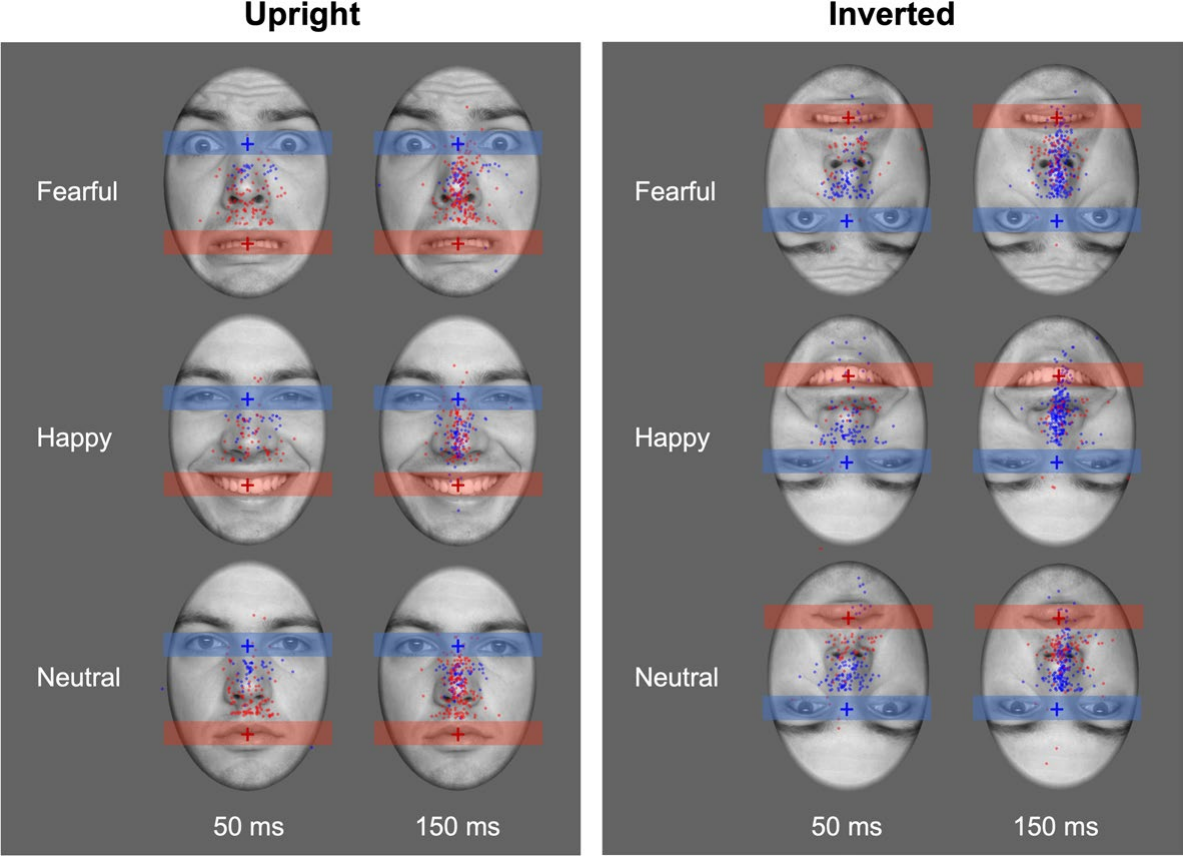
Visual representation of saccade targets across all conditions and facial expressions for Experiment 1. Blue dots denote saccadic endpoints for initial fixations on the eye region and red dots show respective data for initial fixations on the mouth. The shaded areas around eyes and mouth depict the regions wherein saccades were defined as still focussing the initially fixated feature (indicated by a coloured cross) instead of being directed towards the feature in the visual periphery.

Following the procedure of similar studies^7,9,10^, subsequent analyses focused on first saccades occurring between 150 and 1000 ms after stimulus onset. This window was chosen to ensure that all included saccades occurred after face offset, consistent with our pre-registration and prior work (e.g.,^10^). We acknowledge that for the 50 ms condition, this approach may have excluded some earlier post-offset saccades. However, an exploratory analysis indicated that such saccades occurring between 50 and 150 ms were very rare (0.5% of all trials) and including these saccades into the analyses did not change the pattern of results (see supplementary material). In line with our pre-registration, we further defined beforehand that saccades needed to have an amplitude of at least 1° of visual angle in the vertical direction. Smaller changes in gaze position were defined as continuous fixation on the defined facial feature at stimulus onset (see Fig. 2for an illustration of this region). Similar to our previous studies (e.g.,^10^), detected saccades were then classified according to whether they were directed towards the major facial feature that appeared in the periphery. Thus, for example, when an upright face was shown with the initial fixation on the eye region, a downward saccade with a vertical component of at least 1° of visual angle was classified as being directed towards the mouth region. Following this categorization of individuals trials, we calculated the proportion of upward or downward saccades towards the major facial feature in the visual periphery relative to all valid trials in the respective condition, including those in which no saccade occurred or a saccade into the opposite direction was observed, respectively. Note that this calculation differs from the approach used by Atkinson & Smithson^4^, where saccade proportions were computed relative to all trials containing a saccade from a specific fixation location. Since saccades in the opposite direction were exceedingly rare in this experiment (occurring in only 0.65% of trials), the calculated proportions can be interpreted as the probability of switching gaze between facial features rather than maintaining fixation on the initially attended feature.

These proportions were compared between experimental conditions using a 2 × 2 × 3 × 2 repeated measures analysis of variance (ANOVA) with the factors *orientation* (upright, inverted), *presentation time* (50, 150 ms), *emotional expression* (fearful, happy, neutral), and *initial fixation* (eyes, mouth). Based on our hypotheses, we expected to observe two triple interactions: 1) an interaction between presentation time, emotional expression, and initial fixation; 2) an interaction between orientation, emotional expression and initial fixation. To further decompose these potential interactions, we pre-registered follow-up 3 × 2 repeated measures ANOVAs with the factors *emotional expression* and *initial fixation* for upright and inverted faces and for 150 ms and 50 ms *presentation time,* respectively, and expected a significant interaction between emotional expression and initial fixation for upright faces and 150 ms presentation time but statistically less reliable effects for inverted faces and 50 ms presentation time.

#### Behavioural data

Unbiased hit rates were calculated for each condition to examine the influence of the experimental manipulations on emotion classification accuracy. We chose this measure to account for interindividual differences in the frequency of specific emotion classifications. The unbiased hit rate was calculated by dividing the squared frequency of hits (i.e., correct responses) by the product of stimuli showing the respective emotional expression and the frequency of trials where this expression was chosen by the participant^38^. Unbiased hit rates were analysed using a 2 × 2 × 3 × 2 ANOVA with the within subject factors *orientation* (inverted, upright), *presentation time* (50, 150 ms), *emotional expression* (happy, fearful, neutral), and *initial fixation* (eyes, mouth). We expected to observe main effects of stimulus orientation and emotional expression with higher accuracies for upright as compared to inverted and for happy as compared to fearful and neutral facial expressions, respectively. Additional exploratory analyses were conducted on the response times of correct responses (see supplementary material).

#### Statistical analyses

The Greenhouse–Geisser procedure was applied to correct for potential violations of the sphericity assumption in all ANOVAs. The a priori significance level was set to α = 0.05 for all statistical tests. Partial eta squared (*η*_*p*_^*2*^) is reported as a measure of effect size^39,38,41^. To control for potential issues related to multiple comparisons, we applied the Bonferroni-Holm method for all posthoc tests (pairwise and planned comparisons), and report corrected *p*-values.

### Results

#### Proportion of saccades: first saccade after stimulus onset

Figure 1B shows that the overall proportions of saccades as a function of orientation, emotional expression and initial fixation for both presentation times. The ANOVA did not reveal a significant 4-way interaction of all factors, *F*(2, 118) = 2.17, ε = 0.96, *p* = 0.119, η_p_^2^ = 0.04. However, we specifically expected to see two triple interactions and indeed observed a significant interaction between presentation time, facial expression, and initial fixation, *F*(2, 118) = 11.30, ε = 0.88, *p* < 0.001, η_p_^2^ = 0.16, and between orientation, emotional expression, and initial fixation, *F*(2, 118) = 3.81, ε = 0.94, *p* = 0.025, η_p_^2^ = 0.06 (all remaining main and interaction effects are reported in Table S2 in the supplementary material). To further decompose these interactions, separate 3 × 2 repeated measures ANOVAs were conducted for upright and inverted faces and presentation times of 150 and 50 ms, with the factors of emotional expression and initial fixation, respectively.

For upright faces, we observed main effects of emotional expression, *F*(2, 118) = 10.16, ε = 0.96, *p* < 0.001, η_p_^2^ = 0.15, and initial fixation, *F*(1, 59) = 9.10, *p* = 0.004, η_p_^2^ = 0.13, as well as an interaction of both factors when faces were shown for 150 ms, *F*(2, 118) = 15.29, ε = 0.94, *p* < 0.001, η_p_^2^ = 0.21. The latter effect was driven by more saccades towards the eye than the mouth region for fearful and neutral faces (*p*s < 0.006), but not for happy faces (*p* = 1.00). A similar, albeit weaker interaction effect was also observed for a presentation time of 50 ms, *F*(2, 118) = 3.86, ε = 0.98, *p* = 0.024, η_p_^2^ = 0.06. In this case, more saccades towards the eye region than the mouth region were observed for fearful faces (*p* = 0.03) but not for happy or neutral ones (*p*s > 0.09). Similar to the longer presentation time, the main effects of emotional expression, *F*(2, 118) = 9.80, ε = 0.82, *p* = < 0.001, η_p_^2^ = 0.14, and initial fixation, *F*(1, 59) = 7.29, *p* = 0.009, η_p_^2^ = 0.11, also reached significance indicating that across both presentation times, saccades towards the eyes were more frequent than towards the mouth and that overall, happy faces triggered slightly less saccades than the other expressions (see Fig. 1B). Importantly, while saccade direction was clearly modulated by emotional expression—showing a higher proportion of upward shifts toward the eye region for fearful and neutral faces—these saccades often landed short of the feature itself (see Fig. 2 for saccade endpoints).

For inverted faces, we observed a significant main effect of initial fixation, *F*(1, 59) = 39.23, *p* < 0.001, η_p_^2^ = 0.40, and a significant interaction between emotional expression and initial fixation when stimuli were shown for 150 ms, *F*(2, 118) = 13.71, ε = 0.95, *p* < 0.001, η_p_^2^ = 0.19. The main effect of emotional expression was not significant, *F*(2, 118) = 0.07, ε = 0.93, *p* = 0.936, η_p_^2^ = 0.19. As depicted in Fig. 1B, we observed more saccades towards the mouth region (i.e., upwards) than towards the eye region (i.e., downwards) across all facial expressions but this effect was slightly more pronounced for happy and fearful (*p*s < 0.001) than for neutral faces (*p* = 0.005). When presenting inverted faces for only 50 ms, the interaction between facial expression and initial fixation did not reach statistical significance, *F*(2, 118) = 0.02, ε = 0.92, *p* = 0.971, η_p_^2^ < 0.001, but the main effects of emotional expression, *F*(2, 118) = 3.71, ε = 0.89, *p* = 0.033, η_p_^2^ = 0.06, and initial fixation were significant, *F*(1, 59) = 7.98, *p* = 0.006, η_p_^2^ = 0.12. Across all emotional expressions, more saccades occurred for initial fixations on the eye region and irrespective of the initial fixation, neutral faces seemed to trigger more saccades than fearful or happy faces (see Figs. 1B, 2 for an illustration of saccade endpoints). Taken together, gaze orienting for inverted faces differed markedly from that observed for upright faces, suggesting that gaze shifts were not primarily driven by low-level visual features such as local intensity contrasts.

#### Classification performance

The mean unbiased hit rate in the emotion classification task across all experimental manipulations amounted to 0.89. Consistent with our expectations, we obtained significant main effects of emotional expression, *F(*2, 118) = 59.20, ε = 0.69, *p* < 0.001, η_p_^2^ = 0.50, and orientation, *F*(1, 59) = 89.82, *p* < 0.001, η_p_^2^ = 0.60 (for statistics on further effects see Table S3 in the supplementary material). The former effect shows that happy faces were more accurately classified than fearful or neutral ones (*p*s < 0.001), and the latter effect revealed that participants had a higher unbiased hit rate when classifying upright compared to inverted face stimuli (*p* < 0.001), see (Fig. 1C).

### Conclusion

This first experiment examined whether participants show saccades towards diagnostic features of emotional expressions even when faces are shown very briefly (50 ms) as compared to the presentation duration of 150 ms that was often used in previous studies (e.g.,^9,10^). Moreover, we implemented an experimental condition with inverted faces to control for the effects of low-level visual saliency. Across all conditions, saccades occurred only in a minority of trials (approximately 15%). This is not surprising given that with the current brief presentation intervals of 50 and 150 ms, respectively, these saccades do not help to gather more information since the stimuli already disappeared from the screen once a saccade reached its endpoint or even started. Interestingly though, we observed that the pattern of saccades depended on the emotional expression of the presented faces and the initial fixation. Across both presentation durations, we observed that participants made fewer saccades from the mouth to the eye region when viewing happy faces. Saccadic shifts towards the eyes, however, were more prevalent when viewing fearful and neutral expressions. Although these effects were more pronounced for a longer presentation duration, they were also evident when faces were only shown for 50 ms. Since comparable effects were not observed when faces were shown in an inverted orientation, we assume that they indicate a spatial reorientation of attention towards facial features that are diagnostic for specific emotional expressions instead of merely reflecting enhanced attention towards brighter or contrast-rich image regions. Given that face inversion substantially alters these effects, it seems sensible to assume that typical facial configurations support the detection of relevant facial cues in the visual periphery and drive the selection of appropriate targets for upcoming saccades. Concerning the classification performance, we observed that upright faces were classified more accurately than inverted ones and that happy faces seemed to be specifically easy to recognize—even when presented in an inverted fashion^42^. These results are consistent with the so-called “happy superiority” effect and align with previous findings^43,42,45^. Although saccades seem to be preferentially directed at diagnostic facial features, the pattern of classification performance indicates that foveating on these diagnostic features at stimulus onset does not further enhance classification performance. Overall, these results suggest that early saccadic biases occur without necessarily improving behavioural accuracy under brief exposure. It needs to be kept in mind, however, that even under the current highly restricted viewing conditions, emotion recognition performance was at ceiling.

## Experiment 2

This experiment aimed to test another difference in the experimental design of previous studies that found a preferential attentional orienting towards diagnostic features (e.g.,^9,10,33^) and recent studies that failed to observe such effect^4,31^. Specifically, while most earlier studies manipulated the initial fixation using only two potential locations (horizontally centred at the eye- or mouth region, respectively), the latter studies used six possible locations including positions at the left or right eye and the left or right cheek in addition to the centre of the mouth and the glabella between the eyebrows. In Experiment 2, we directly compared these two conditions. Thus, participants completed one experimental block with only two possible locations of the initial fixation and a second block with six possible locations. Based on the results of Atkinson and Smithson^4^ and Duran and Atkinson^31^, we expected to find weaker effects of an attentional orienting towards diagnostic facial features in the latter condition. Regarding the behavioural data, we again assumed a more accurate classification of happy expressions and exploratively analysed the effect of the other experimental manipulations on classification accuracy and response times.

### Methods

#### Participants

Based on the comparably large effects obtained in Experiment 1, we pre-registered a sample size of 40 participants for this experiment. A simulation^32^ using the data of Experiment 1 indicated a power of ~ 0.75 for detecting a three-way interaction between experimental block, emotional expression, and initial fixation (see supplementary material for further details).

We recruited a total of 49 participants via an online database for psychological studies of the University of Würzburg, but 5 participants had to be excluded due to less than 70% valid trials (see below). This resulted in a final sample of 44 participants (36 female) aged 19–33 years (*M* = 23.7 years, *SD* = 2.9 years) who reported normal or corrected-to-normal vision. This exceeds the sample size of 40 participants that was pre-registered, but we decided to analyse all available data. This increases the power for detecting the three-way interaction to ~ 0.80 and restricting the analyses to the data of the first 40 valid participants does not change the pattern of results reported below. All participants provided written informed consent and either received course credits or were compensated with 10 €.

#### Stimuli and design

We used the same stimulus set as in Experiment 1. From this set, 96 images per emotional expression (48 women, 48 men) were randomly selected for each participant and an additional set of 8 images for each emotional expression was randomly selected for a short training block to familiarize participants with the task.

The selected stimuli were assigned to two experimental conditions. One block with only two potential initial fixations and the second block with six potential initial fixations. The first block represented a 3 × 2 design with the factors *emotional expression* (fearful, happy, neutral) and *initial fixation* (eyes, mouth) with 18 trials for each factor combination (nine women, nine men), amounting to 108 trials in total. The second block was a 3 × 6 design with the factors *emotional expression* (fearful, happy, neutral) and *initial fixation* (left eye, glabella, right eye, left cheek, centre of the mouth, right cheek) with 10 trials for each factor combination (five women, five men), amounting to 180 trials in total which were split into two sessions with 90 trials each.

#### Apparatus

The study was programmed using the software Presentation (Neurobehavioral Systems, Berkeley, CA, USA) and stimuli were displayed on a 24" Asus VG248QE monitor (53.14 × 29.89 cm) with a resolution of 1920 × 1080 pixels and a refresh rate of 144 Hz. Due to the 144 Hz refresh rate, the actual face presentation duration (nominally 150 ms) corresponds to either 21 or 22 frames, that is, approximately 146 or 153 ms. While minor, this constraint is inherent in frame-based presentation systems and should not affect the current results. Similar to Experiment 1, participants were placed in a soundproof booth with dim light, with eye movements of the right eye tracked using an EyeLink 1000 Plus system (SR Research, Ottawa, ON, Canada). Eye-tracking data were recorded at a sampling rate of 1000 Hz. A desktop setup with a chin rest and a forehead bar was used with a distance of 55 cm between the eyes of the participants and the centre of the screen. Under these conditions, the visual angle of the stimuli amounted to approximately 14.12° × 19.98°.

#### Procedure

The procedure was largely identical to Experiment 1 and included the same COVID-19 safety measures and a training block with 24 trials at the beginning to familiarize participants with the task and the assignment of keys on the computer keyboard to emotional expressions. Following the training block, participants accomplished the main experiment with the order of the experimental conditions (i.e., two vs. six possible initial fixations) counterbalanced across participants. After each block, participants were allowed a short break.

The trial structure was largely identical to Experiment 1. In short, a fixation cross at the centre of the screen was shown for 500 ms, followed by the face stimulus for 150 ms and a blank screen for 1850 ms. Faces were unpredictably shifted horizontally and vertically to manipulate which facial feature would be presented at the location of the formerly shown fixation cross. During the intertrial interval (random duration between 1000 and 3000 ms), the fixation cross was shown again.

### Data processing and analysis

#### Eye-tracking data

Data were processed similarly as in Experiment 1 to detect saccades that were triggered by the face presentation. Applying the same criteria to identify invalid trials (i.e., outliers in baseline coordinates or blinks between -300 and 150 ms relative to stimulus onset) resulted in the exclusion of 5 participants due to less than 70% of valid trials (see participants section above). In the final sample, 10.1% of trials were excluded on average (*SD* = 7.4%).

Similar to Experiment 1, we calculated the proportion of trials with saccades that were directed towards the facial feature that was presented in the periphery relative to all valid trials of the respective condition (i.e., including saccades into the opposite direction as well as continuous fixations). Corresponding to Experiment 1, saccades towards the facial outline (e.g., upwards from the eye region) were exceedingly rare and only occurred in 0.54% of all trials. To allow for a comparison between experimental blocks, we calculated the proportion of downward saccades exceeding an amplitude of 1° of visual angle when the initial fixation was in the upper part of the face and the proportion of upward saccades when the cheeks or mouth were presented at fixation. Smaller changes in gaze position were defined as continuous fixation on the defined facial feature at stimulus onset (see Fig. 5 for an illustration of this region). Please note that we also planned a more fine-grained analysis of saccade targets using the projection measures described by Atkinson and Smithson^4^ but unfortunately, several participants did not show any saccade in specific experimental conditions, resulting in missing values for the saccade projection measure. Since this would entail losing about half of the sample for the main analyses, we decided to concentrate on the analyses of the proportion of saccades as a function of the experimental conditions.

In a first step, saccade proportions were compared between experimental blocks using a 2 × 3 × 2 repeated measures ANOVA with *experimental block* (two vs. six initial fixation locations), *emotional expression* (fearful, happy, neutral), and *initial fixation* (eye region/glabella, mouth region) as factors. To further characterize the pattern of saccades in each block, we also calculated separate 3 × 2 (block 1) and 3 × 6 (block 2) ANOVAs within each block using *emotional expression* (fearful, happy, neutral), and *initial fixation* (two or six positions) as factors. To further decompose interaction effects, pairwise t-tests were conducted to assess whether significant differences existed in the proportion of saccades toward the eyes compared to the mouth across left, middle, and right initial fixation locations for each facial expression. Cohen’s *d* was calculated to quantify the effect size of these differences.

#### Behavioural data

Similar to Experiment 1, analyses of behavioural data focused on unbiased hit rates that were first compared between experimental blocks using a 2 × 3 × 2 repeated measures ANOVA with *experimental block* (two vs. six initial fixation locations), *emotional expression* (fearful, happy, neutral), and *initial fixation* (eye region/glabella, mouth region) as factors. Afterwards, we also calculated separate 3 × 2 (block 1) and 3 × 6 (block 2) ANOVAs within each block using *emotional expression* (fearful, happy, neutral), and *initial fixation* (two or six positions) as factors. Additional exploratory analyses were conducted on the response times of correct responses (see supplementary material).

#### Statistical analyses

All statistical procedures were identical to Experiment 1.

### Results

#### Proportion of saccades: first saccade after stimulus onset

In a first step, we analysed differences between blocks by calculating an ANOVA on the proportion of first saccades with block, emotional expression and initial fixation as factors. Most importantly, this analysis revealed a significant interaction of all three factors, *F*(2, 86) = 5.26, ε = 0.97, *p* = 0.007, η_p_^2^ = 0.011 (all remaining main and interaction effects are reported in Table S6 in the supplementary material). To follow up on these differences between blocks, we conducted separate ANOVAs for block 1 (two possible initial fixations) and block 2 (six possible initial fixations).

**Fig. 3:**
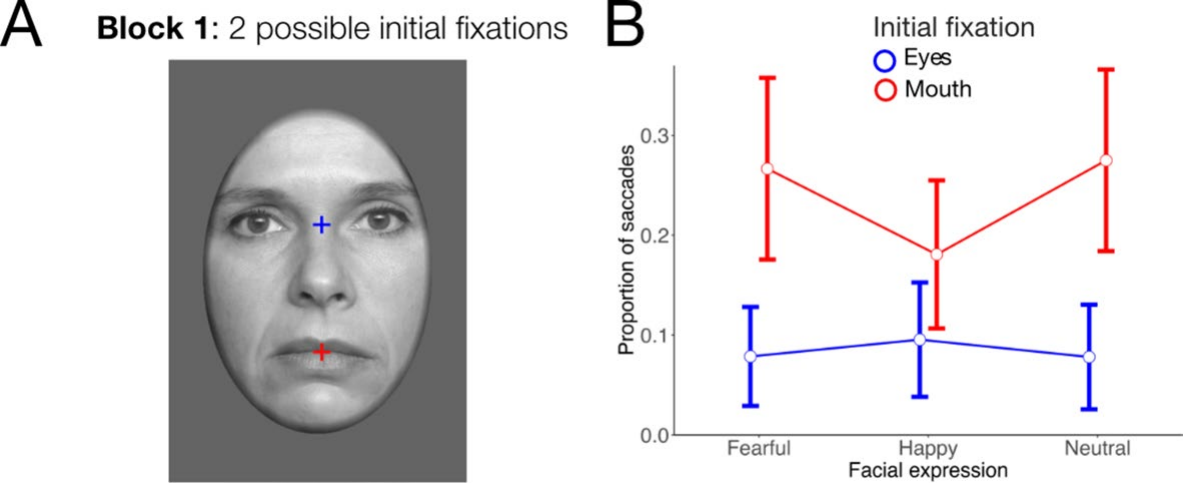
Depiction of the experimental manipulation and corresponding results for the two initial fixation locations in block 1 of Experiment 2. (**A**) Locations of initial fixations on the eye and mouth region, respectively, with colours used solely for illustrative purposes. (**B**) Proportion of saccades as a function of the emotional expression and initial fixation location. Error bars represent 95% confidence intervals.

The ANOVA on the proportion of saccades in block 1 revealed significant main effects of emotional expression, *F*(2, 86) = 5.97, ε = 0.96, *p* = 0.004, η_p_^2^ = 0.12, and initial fixation, *F*(1, 43) = 11.88, *p* = 0.001, η_p_^2^ = 0.22, as well as a significant interaction of both factors, *F*(2, 86) = 10.32, ε = 0.97, *p* < 0.001, η_p_^2^ = 0.19. As shown in Fig. 3B, the proportion of gaze shifts towards the eye region was greater than towards the mouth region for fearful and neutral facial expressions (*p*s < 0.006) but there was no significant difference between the eye and mouth regions for happy expressions (*p* = 1.00).

**Fig. 4:**
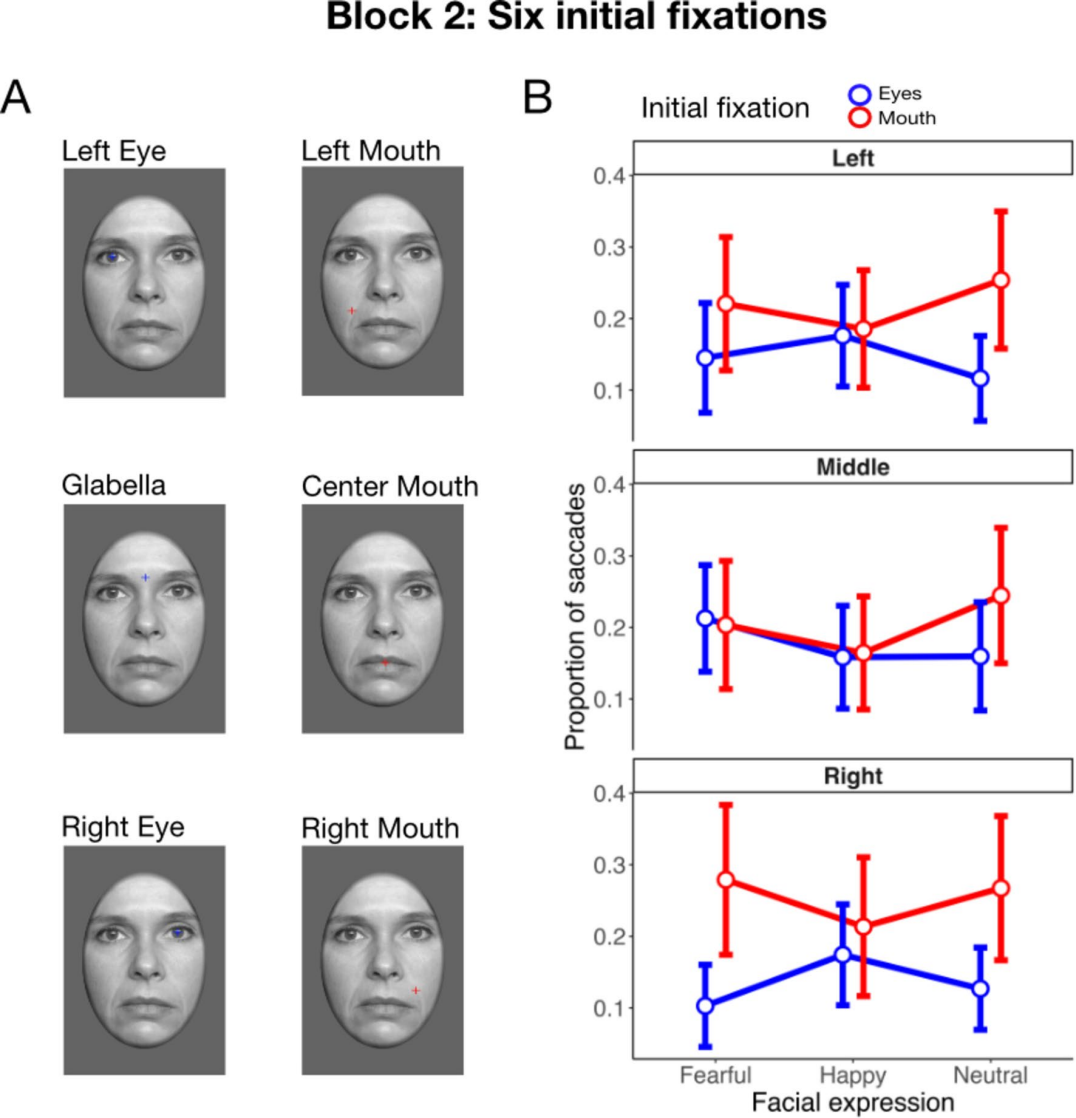
Depiction of the experimental manipulation and corresponding results for the six initial fixation locations in block 2 of Experiment 2. (**A**) Illustration of the six initial fixation locations with colours used solely for illustrative purposes. (**B**) Proportion of saccades as a function of the emotional expression and initial fixation location for the left, middle, and right regions of the face. Error bars represent 95% confidence intervals.

**Fig. 5:**
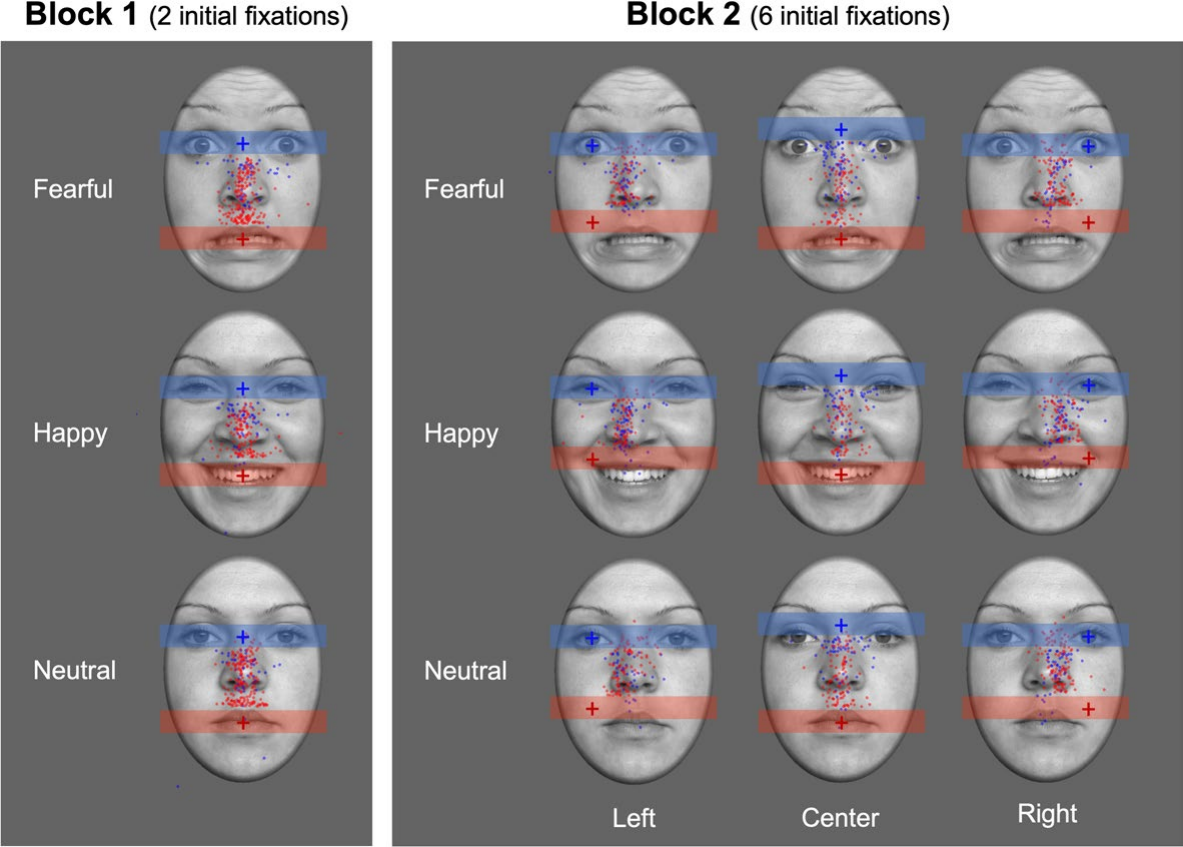
Visual representation of saccade targets across all conditions and facial expressions for Experiment 2. Blue dots denote saccadic endpoints for initial fixations on upper facial regions (i.e., left eye, central eye region/glabella, right eye) and red dots show respective data for initial fixations on lower facial regions (i.e., left cheek, centre of the mouth, right cheek).

For block 2 with six possible initial fixation locations (see Fig. 4A), both main effects failed to reach statistical significance (emotional expression: *F*(2, 86) = 1.72, ε = 0.99, *p* = 0.185, η_p_^2^ = 0.04; initial fixation location: *F*(5, 215) = 2.10, ε = 0.29, *p* = 0.144, η_p_^2^ = 0.05) but we observed a significant interaction of both factors, *F*(10, 430) = 6.18, ε = 0.72, *p* < 0.001, η_p_^2^ = 0.13. To further decompose this interaction, we separately analysed differences between the proportion of upward saccades towards the eyes and downward saccades towards the mouth across the left, middle, and right initial fixation locations for all facial expressions. Interestingly, significant differences were mainly observed for the lateral initial fixation positions and were restricted to fearful and neutral facial expressions (see Table 1). For these conditions, the pattern of results largely resembled the data of block 1 with two possible initial fixation locations (see Figs. 3A,B, 4B). For central initial fixation positions, proportions of saccades were comparable to these conditions for initial fixations on the mouth but deviated for initial fixations on the eye region/glabella. When participants initially fixated on the glabella, they showed comparably more downward saccades. However, these saccades were mostly not targeting the mouth region but were rather oriented towards the eye level (see Fig. 5).

**Table 1:** Pairwise t-test comparisons of the proportion of upward saccades when initially fixating at the mouth region and downward saccades when initially fixating at the eye region for different emotional expressions (fearful, happy and neutral) and initial fixation locations (left, middle, right) in Experiment 2.

	Initial fixation location					
	Left		Middle		Right	
	*t*(43)	*d*	*t*(43)	*d*	*t*(43)	*d*
Fearful	1.18	0.26	0.21	0.03	2.72**	0.62
Happy	0.17	0.04	0.13	0.02	0.60	0.14
Neutral	2.24*	0.51	1.74	0.29	2.23*	0.51

The table presents t-statistics with degrees of freedom in parenthesis and Cohen’s *d* effect size to quantify the difference, *p < .05, **p < .01.

#### Classification performance

Similar analyses as for the saccade data were also performed on unbiased hit rates. The first ANOVA comparing similar fixation positions across blocks yielded no significant effects (see Table S7 in the supplementary material). Separate analyses for each block revealed significant main effects of emotional expression in block 1, *F*(2, 86) = 4.56, ε = 0.79, *p* = 0.020, *η*_*p*_^*2*^ = 0.10, and block 2, *F*(2, 84) = 5.55, ε = 0.89, *p* = 0.007, *η*_*p*_^*2*^ = 0.12 (all other effects failed to reach statistical significance, see Tables S8 and S9 in the supplementary material). These effects mainly indicate highest unbiased hit rates for happy and lowest for fearful facial expressions.

### Conclusion

Consistent with Experiment 1 and previous research in the field^10,46^, Experiment 2 showed that saccades only occurred in a minority of trials (approximately 18% across all conditions) but were modulated by the experimental manipulations. Specifically, participants generally oriented their gaze more often towards the eyes than towards the mouth region for fearful and neutral facial expressions. However, this effect was weaker when six instead of two possible initial fixation locations were used. Detailed analyses of the eye-tracking data of block 2 indicated that the pattern of results was consistent to block 1 (i.e., the experimental condition with only two possible initial fixations) for locations on the left or right side of the face. The main difference emerged for initial fixations on the glabella in block 2. In this case, more downward saccades were observed, effectively reducing differences between initial fixations across emotional expressions. Interestingly, however, these downward saccades did not target the mouth region but rather ended at or around the eyes for fearful and neutral expressions (see Fig. 5B). Thus, they also seemed to be oriented towards facial features that are diagnostic of emotional expressions. Finally, the results indicated that participants were better at recognising happy faces than fearful expressions, which is consistent with previous findings of the “happy superiority effect”^47,46,49^. Similar to the upright presentation in Experiment 1, recognition accuracy did not differ significantly between initial fixation locations which might be due to a ceiling effect in recognition performance.

## General discussion

The current study aimed to investigate the stability of attentional orienting towards diagnostic features of emotional facial expressions. Across two experiments, participants were asked to recognize briefly presented fearful, happy, and neutral facial expressions while controlling for the initial fixation. Emotion recognition performance was quantified using the unbiased hit rate and eye-tracking was used to measure saccades that were triggered by the presented faces. With the current short presentation times, these saccades occur after the stimulus already disappeared from the screen and thus they do not allow for further stimulus exploration^10^.

Previous studies have shown that such reflexive first saccades tended to be oriented towards emotion-distinguishing features such as eyes and mouth, indicating extrafoveal processing of these features. Thus, several studies reported saccades to preferentially target the eye region for fearful faces and the mouth region for happy facial expressions^7–9^). However, recent studies failed to replicate such effect^4,31^. This discrepancy could be due to differences in methodological approaches, or it might indicate the involvement of additional mechanisms, such as a ‘center-of-gravity’ (COG) effect, where saccades tend to land in central, balanced locations on the face, regardless of the emotional expression^58,59^. Such a mechanism could potentially mask emotion-specific saccadic patterns. To elucidate this discrepancy, we systematically examined the influence of two critical experimental manipulations on the pattern of visual orienting in the current study. First, we manipulated the presentation time in Experiment 1, and second, we examined the influence of the number of potential initial fixations in Experiment 2. Furthermore, to account for the effects of low-level visual properties on attentional orienting, we used inverted faces as a control condition in Experiment 1. Across both experiments, each visual stimulus was presented uniquely in a single trial to each participant, thereby eliminating any confounding effects of repeated exposure.

Across both experiments, we replicated the previously observed preferential attentional orienting towards diagnostic facial features, even for brief 50 ms presentations, and it was consistent across initial fixation locations on the left and right sides of the faces (i.e., cheeks and eyes). The choice of exposure durations was driven by the need to limit voluntary exploration and capture rapid orienting. As in previous work^10^, the 150 ms duration ensures that the stimulus offset typically occurs before the initiation of saccades, thus effectively isolating reflexive gaze shifts. The 50 ms condition further tested whether these effects persist under even more constrained visual input. While these timings allow for a precise investigation of reflexive attentional processes, they capture only a narrow window of early face perception. In real-world interactions, emotion recognition typically unfolds over longer durations and involves reciprocal gaze, motion, and contextual cues. Nevertheless, automatic processes appear to contribute to gaze regulation in social contexts, as evidenced by impaired eye contact in neuropsychological cases^50^ and in individuals with autism spectrum disorder^51^—likely driven by difficulties in early attentional orienting^52^. Future research employing more naturalistic paradigms is needed to clarify how early orienting interacts with attentional processes at later stages of emotion perception.

Interestingly, Experiment 1 demonstrated that face inversion disrupts saccadic orienting towards diagnostic features, consistent with a reliance on extrafoveal processing that compares peripheral features to stored face templates for guiding attention^53,54^. This pattern also aligns with the role of the amygdala in modulating attention towards emotionally salient facial features, particularly the eyes of fearful faces. Prior studies have shown that amygdala activation enhances gaze shifts toward fearful eye regions^9^, and that this bias is diminished in individuals with amygdala lesions^55^. Such findings support the view that the reflexive gaze shifts observed here may be amygdala-driven and routed via subcortical structures (e.g., superior colliculus—pulvinar—amygdala pathway; see^56^), especially when holistic face perception is intact. Future studies employing high-resolution neuroimaging or investigations with neuropsychological patients should further clarify whether the attentional effects observed here are truly linked to the hypothesized neural circuitry, and whether they are emotion-specific or reflect a general mechanism for attending to diagnostic facial features.

Notably, our results differ from previous studies that found no variation in fixation locations across emotional expressions^5,14^, likely due to our shorter presentation times (≤ 150 ms) and controlled initial fixations, which emphasized rapid, reflexive saccades towards diagnostic features. The only divergence in our findings occurred in Experiment 2 with central fixation presentations, where fixations at the glabella led to more downward saccades, yet these still targeted the eye region, particularly in fearful and neutral faces. Thus, even in this setup—that matched the typical experimental paradigm of the studies by Atkinson and colleagues^4,31^—we observed evidence for a saccadic orienting towards emotion-distinguishing features.

A closer inspection of saccade endpoints also shows that saccades typically do not reach the eye or mouth region but rather land around the centre of the face. Such central tendency has often been reported^57,58^ and is commonly attributed to a ‘centre-of-gravity’ (COG) effect, which may reflect a default strategy for optimising future exploration^59^. In our study, this tendency persisted even when visual exploration was precluded by brief exposure, but it was modulated by the emotional expression. While we observed feature-specific saccade biases for many conditions, the increased downward saccades from glabella fixations in Experiment 2 suggest a complex interplay between diagnostic feature selection and COG-based anchoring. These downward saccades did not land on the mouth but often near the eyes, especially for fearful and neutral faces (see Fig. 5), indicating that early gaze shifts are shaped by both emotional relevance and spatial regularities of face structure.

The fact that saccadic biases also occurred in the current study, where visual exploration was impossible due to very short presentation times, indicates that this reorienting occurs automatically. Interestingly, however, such centring did not occur unselectively across all stimuli but was modulated by the emotional expression. Thus, when the initial fixation was on a facial feature that was less diagnostic for the depicted emotional expression, more saccades left this feature towards the other major facial feature in the visual periphery. Conversely, when the initial fixation was on a facial feature that was diagnostic for the currently displayed emotional expression, saccades were rather inhibited. Thus, saccades seemed roughly oriented towards diagnostic features but did not seem to be precisely directed towards them. This might explain why detailed analyses of saccadic trajectories, that were unfortunately not possible in the current study due to missing data, did not find evidence for a specific orientation towards emotion-distinguishing facial features^4,31^. However, the currently presented coarse analyses of saccade directions already provides evidence for a significant effect of emotional expressions on extrafoveal processing and visual reorienting.

Although it might seem plausible to assume that fixating a diagnostic feature enhances emotion recognition accuracy, this effect was only inconsistently observed in previous studies^4,10,11,33,55^, and was not supported in our current data. Importantly, our findings are limited to fear, happiness, and neutral expressions. Emotions such as anger, disgust, or surprise—which differ in feature distribution and ambiguity—may engage different mechanisms. As Atkinson & Smithson^4^ suggested, the effect of enforced fixation on recognition accuracy may strongly depend on the particular emotion set tested. This remains an open empirical question, and future studies directly comparing different sets of emotional expressions under controlled conditions would be highly informative. In the current two experiments, we only observed a significantly worse performance when faces were inverted and when they were only shown for 50 ms. Interestingly, however, even under such restricted or suboptimal viewing conditions, classification performance was very good. Such ceiling effect along with substantial individual difference in face exploration^60^ might have contributed to previous difficulties of observing a significant effect of foveal processing of specific facial features on emotion recognition. Across both experiments, we observed higher classification accuracies of happy facial expressions. This finding reflects the “happy superior effect”^61^ and has frequently been observed before^3,33^. For future research, it seems very interesting to further restrict viewing conditions to enable a better differentiation between the emotion recognition performance across experimental conditions. For example, this can be achieved by degrading the visual stimuli (e.g., using noise masks) or by introducing a second demanding task that requires attentional resources.

Although the current study provides important insight into the stability of attentional orienting towards emotion-distinguishing facial features, there are several limitations that should be considered for future research. First, as already noted, it would be valuable to include additional facial expressions in future studies (e.g., anger, sadness, disgust). This would provide a more comprehensive understanding of the generalizability of the current findings on the processing of emotional expressions. Secondly, the use of congruent and incongruent faces could help to further probe the role of diagnostic features for accurate emotion classification. Incongruent faces, where the upper and lower parts of the face are composed of different facial expressions, are known to inhibit holistic face processing and instead promote feature-based strategies. Such manipulation could therefore provide a more nuanced understanding of how different diagnostic features contribute to face perception and emotion recognition (see^62^). Finally, future studies should explore individual differences in facial exploration^60^ and emotion recognition^63^ in more detail and combine these findings with experimental paradigms that allow for dissecting reflexive and more sustained attentional processes in face processing that might rely on different neurocognitive systems^56^. Future work could also integrate eye-tracking with EEG-based multivariate pattern analysis (MVPA) to clarify whether emotional differentiation occurs prior to gaze shifts. These approaches offer higher temporal resolution than traditional studies of face-sensitive components of event-related brain potentials and may help clarify whether early categorical encoding of facial expressions precedes reflexive gaze shifts towards diagnostic features.

## Conclusion

To sum up, the current study demonstrated a robust tendency to automatically orient the gaze towards emotionally diagnostic facial features. This effect occurred even with presentation times as brief as 50 ms and was relatively stable across different initial fixation positions. These findings indicate an extrafoveal processing of facial features and the substantially altered pattern for inverted faces suggests a continuous comparison of current visual input to internal face templates for the programming and initiation of saccades. Face recognition was unaffected by the foveated feature which might be due to ceiling effects in recognition performance, individual differences or a holistic rather than a feature-based processing of facial expressions. Future studies should elucidate to what degree these findings also generalize to other facial expressions and viewing conditions.

## Supplementary Information

Supplementary Information.

## Data Availability

Trial-wise data for this study are available in the Open Science Framework (OSF) repository at https://osf.io/g2sk9/. These data along with the posted scripts allow for reproducing the analyses reported in this article.

## Declarations

### Public significance statement

This research reveals that individuals automatically shift their attention to facial features that are important to understand the emotional state of conspecifics. Focusing on these regions does however not necessarily improve emotion recognition abilities.
