# Supplementary material for: Facial features associated with fear and happiness attract gaze during brief exposure without enhancing emotion recognition
Source: Sci Rep. 2025 Aug 19;15:30442. doi: 10.1038/s41598-025-12327-6 (PMC12365274; doi:10.1038/s41598-025-12327-6)
Supplement: Supplementary file 1 — Supplementary Information. [file 41598_2025_12327_MOESM1_ESM.pdf]

Supplementary information

Facial Features Associated with Fear and Happiness Attract Gaze During Brief Exposure Without Enhancing Emotion Recognition

Yu-Fang Yang*[1,2], Matthias Gamer[1]

[1]Department of Psychology, University of Würzburg, Würzburg, Germany

[2]Division of Experimental Psychology and Neuropsychology, Department of Education and Psychology, Freie Universität Berlin, Berlin, Germany

Experiment 1

Power analysis

For sample size calculations, we relied on a simulation [1] using estimates of *M*, *SD*, and the correlation between factor levels from the largest of our previous studies ($N = 79$[2]) that used a very similar design as the current experiment. For the simulation, we relied on the proportion of saccades that was observed in the previous study since our main hypotheses focused on the eye-tracking data. We simulated a pattern of expected effects for a 2 × 3 × 2 interaction consistent with our hypotheses (i.e., interactions between *orientation*, *emotional expression*, and *initial fixation* as well as between *presentation time*, *emotional expression*, and *initial fixation*; see Table S1). The common *SD* was estimated at 0.21 and the correlation between factor levels at .55. Using the Greenhouse-Geisser sphericity correction at an a priori significance level of $\alpha = .05$ in 2000 simulations revealed a power of $1 - \beta = .805$ for detecting the triple interaction in a sample size of $N = 60$ participants.

Table S1. Assumed effect structure for the power analysis simulations that were used to calculate the sample size for Experiment 1.

Emotional expression	Initial fixation	Presentation time 150 ms / Upright presentation	Presentation time 50 ms / Inverted presentation
Fearful	Eyes	0.19	0.19
	Mouth	0.45	0.44
Happy	Eyes	0.18	0.19
	Mouth	0.36	0.44
Neutral	Eyes	0.20	0.19
	Mouth	0.51	0.44

Note. For the newly introduced manipulations, the average effect of the previously observed saccadic proportions was assumed.

Table S2. Results of the ANOVA on the proportion of saccades using the within-subject factors presentation duration, orientation, emotional expression, and initial fixation.

Effect	*df1*	*df2*	ε_{GG}	*F*	*p*	η_p^2
Presentation duration	1	59	-	69.60	< .001***	.03
Orientation	1	59	-	8.17	.006**	.04
Emotion	2	118	.084	9.39	< .001***	.00
Initial fixation	1	59	-	3.07	.085	.01
Presentation duration × Orientation	1	59	-	1.67	.201	.00
Presentation duration × Emotion	1	188	.863	1.01	.366	.00
Orientation × Emotion	2	118	.963	6.74	.002**	.01
Presentation duration × Initial fixation	1	59	-	5.38	.024*	.02
Orientation × Initial fixation	1	59	-	25.23	< .001***	.08
Emotion × Initial fixation	2	118	.866	18.09	< .001***	.02
Presentation duration × Orientation × Emotion	2	118	.929	1.02	.363	.00
Presentation duration × Orientation × Initial fixation	1	59	-	28.71	< . 001***	.00
Presentation duration × Emotion × Initial fixation	2	118	.878	11.30	< .0001***	.00
Orientation × Emotion × Initial fixation	2	118	.938	3.81	.025**	.00
Presentation duration × Orientation × Emotion × Initial fixation	2	118	.964	2.17	.119	.00

Note. *** $p < .001$, ** $p < .01$, * $p < .05$

Table S3. Results of the ANOVA on unbiased hit rates using the within-subject factors presentation duration, orientation, emotional expression, and initial fixation.

Effect	*df1*	*df2*	ε_{GG}	*F*	*p*	η_p^2
Presentation duration	1	59	-	5.28	.025*	.01
Orientation	1	59	-	89.82	<.001***	.60
Emotion	2	118	.688	59.20	<.001***	.50
Initial fixation	1	59	-	8.08	.006**	.12
Presentation duration × Orientation	1	59	-	1.54	.220	.03
Presentation duration × Emotion	2	118	.971	0.65	.523	.01
Orientation × Emotion	2	18	.960	32.19	<.001***	.35
Presentation duration × Initial fixation	1	59	-	3.31	.074	.05
Orientation × Initial fixation	1	59	-	3.11	.083	.05
Emotion × Initial fixation	2	118	.809	7.85	<.001***	.12
Presentation duration × Orientation × Emotion	2	18	.859	0.23	.791	.00
Presentation duration × Orientation × Initial fixation	1	59	-	2.05	.157	.03
Presentation duration × Emotion × Initial fixation	2	118	.855	4.12	.019**	.07
Orientation × Emotion × Initial fixation	2	118	.881	9.28	<.001***	.14
Presentation duration × Orientation × Emotion × Initial fixation	2	118	.969	2.06	.132	.03

Note. *** $p < .001$, ** $p < .01$, * $p < .05$.

Reaction Times

The 2 × 2 × 3 × 2 ANOVA on reaction times of correct responses in the emotion recognition task using the within-subject factors presentation duration, orientation, emotional expression, and initial fixation revealed main effects of stimulus orientation, ($F(1, 59) = 100.74$, $p < .001$, $\eta_p^2 = .63$), facial expression, $F(2, 118) = 55.14$, $\varepsilon = .97$, $p < .001$, $\eta_p^2 = .48$), and initial fixation location, ($F(1, 59) = 26.33$, $p < .001$, $\eta_p^2 = .31$). Furthermore, significant interaction effects were observed for presentation duration × orientation, ($F(1,59) = 14.60$, $p < .001$, $\eta_p^2 = .20$), presentation duration × emotional expression, ($F(2,118) = 7.92$, $\varepsilon = 1.00$, $p < .001$, $\eta_p^2 = .12$), stimulus orientation × emotional expression, ($F(2,118) = 12.88$, $\varepsilon = .92$, $p < .001$, $\eta_p^2 = .18$), and emotional expression × initial fixation, ($F(2,118) = 15.96$, $\varepsilon = .92$, $p < .001$, $\eta_p^2 = .21$). All remaining main and interaction effects were not statistically significant (see Figure S1).

Overall, participants were faster for upright as compared to inverted faces and for happy as compared to fearful and neutral facial expressions, but these effects were more pronounced for short (50 ms) as compared to longer presentation durations (150 ms). Face inversion had a stronger effect on fearful and neutral facial expressions as compared to happy faces. Finally, response times were comparable between initial fixation locations for fearful and happy facial

expressions, but participants were slower in recognizing neutral facial expressions when initially foveating on the eye region.

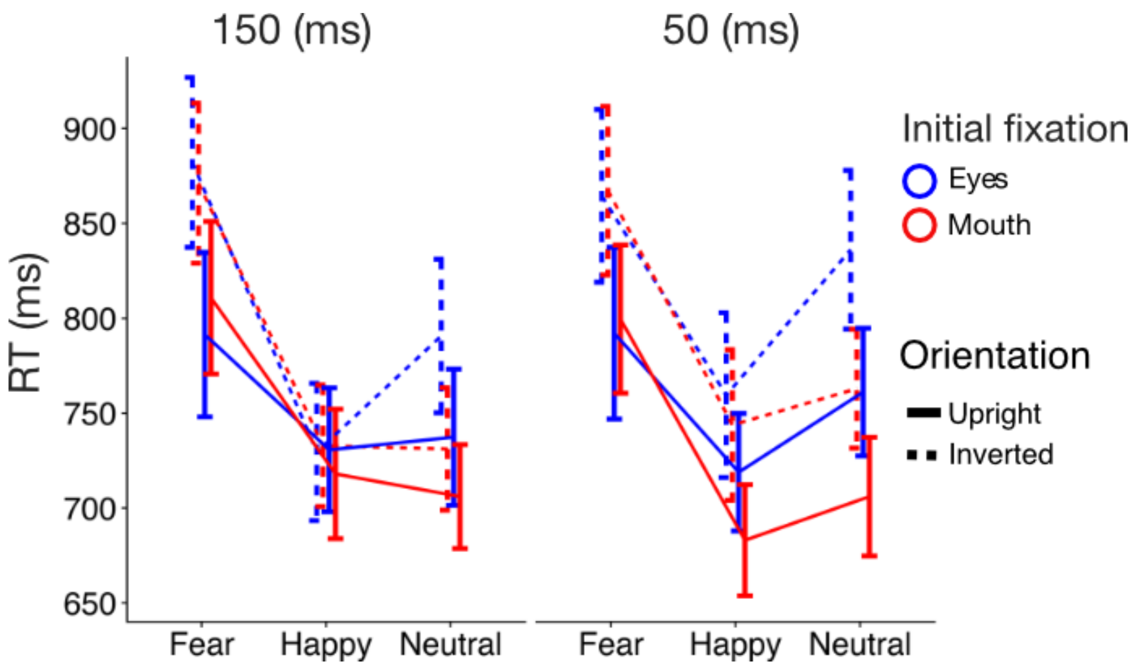

Figure S1: Reaction times (RTs) for initial fixations on the eyes (blue) and mouth (red) across facial expressions under different orientation and presentation durations. RTs are depicted for fearful, happy, and neutral expressions presented for 150 ms (left) and 50 ms (right). Solid lines indicate an upright and dashed lines an inverted presentation, respectively. Error bars represent 95% confidence intervals.

Inclusion of earlier saccades

The pre-registered analyses reported in the article relied on a fixed analysis window of 150 to 1000 ms. However, in the short presentation time condition, faces disappeared after just 50 ms, potentially allowing for earlier saccades that may have been missed with the original analysis window. Although saccades occurring between 50 and 150 ms were rare – accounting for only 0.5% of all trials – we conducted additional analyses to assess whether including these early saccades would affect the results. To maintain consistency in scoring windows, we applied a 50 to 900 ms window for the short presentation duration (50 ms) and retained the original 150 to 1000 ms window for the longer presentation (150 ms). As shown in Table S4, the pattern of significant effects closely mirrored the original analyses (see Table S2). Furthermore, Figure S2 demonstrates that incorporating the few early saccades does not alter the overall pattern of results (cf. Figure 1B for the original analyses).

Table S4: Results of the ANOVA on the proportion of saccades using the within-subject factors presentation duration, orientation, emotional expression, and initial fixation. Note that different time windows were applied for saccade detection for 50 ms and 150 ms presentation duration, respectively.

Effect	*df1*	*df2*	ε_{GG}	*F*	*p*	η_p^2
Presentation duration	1	59	-	74.73	< .001***	.29
Orientation	1	59	-	9.11	.004**	.01
Emotion	2	118	0.84	9.79	< .001***	.03
Initial fixation	1	59	-	3.29	.075	.01
Presentation duration × Orientation	1	59	-	1.28	.262	.00
Presentation duration × Emotion	2	118	0.84	0.73	.483	.00
Orientation × Emotion	2	118	0.96	8.00	.001**	.02
Presentation duration × Initial fixation	1	59	-	4.82	.032*	.01
Orientation × Initial fixation	1	59	-	25.35	< .001***	.08
Emotion × Initial fixation	2	118	0.87	18.24	< .001***	.03
Presentation duration × Orientation × Emotion	2	118	0.96	0.66	.519	.00
Presentation duration × Orientation × Initial fixation	1	59	-	32.40	< .001***	.10
Presentation duration × Emotion × Initial fixation	2	118	0.90	10.40	< .001***	.02
Orientation × Emotion × Initial fixation	2	118	0.91	4.30	.016*	.01
Presentation duration × Orientation × Emotion × Initial fixation	2	118	0.99	2.50	.086	.00

Note. *** $p < .001$, ** $p < .01$, * $p < .05$.

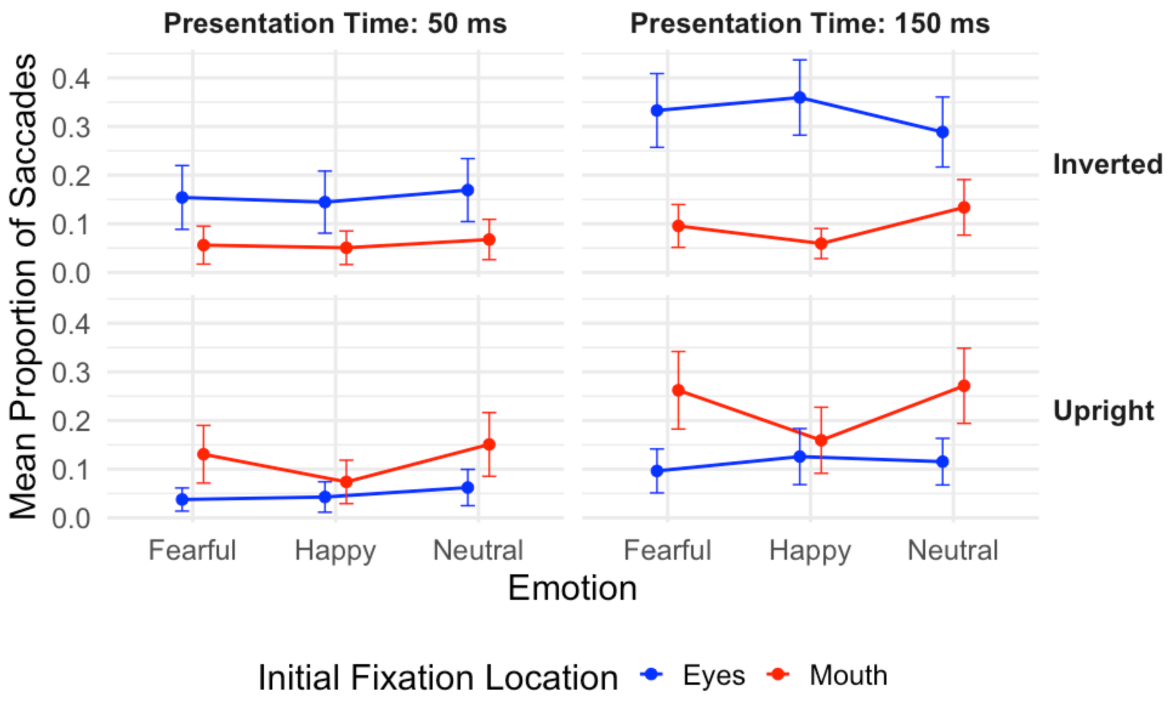

Figure S2: Mean saccade proportions as a function of emotional expression, initial fixation location, orientation, and presentation time. Error bars show 95% confidence intervals.

Experiment 2

Power analysis

For sample size calculations, we again relied on a simulation (Lakens & Caldwell, 2021). However, since the effects were relatively large in Experiment 1 and since a very similar setup and design was used for Experiment 2, values of *M*, *SD*, and the correlation between factor levels were derived from the eye-tracking data of the first experiment. We again constructed a pattern of expected effects for a 2 × 3 × 2 interaction consistent with our hypothesis that for comparable initial fixation locations, we would find a reduced interaction of *emotional expression* and *initial fixation* in the block with six as compared to the block with two initial fixation locations (see Table S4). The common *SD* was estimated at 0.21 and the correlation between factor levels at .61. Using the Greenhouse-Geisser sphericity correction at an a priori significance level of $\alpha = .05$ in 2000 simulations revealed a power of $1 - \beta = .748$ for detecting the triple interaction in a sample size of $N = 40$ participants. Increasing the sample size to $N = 44$ participants raises the power to $1 - \beta = .800$.

Table S5. Assumed effect structure for the power analysis simulations that were used to calculate the sample size for Experiment 2.

Emotional expression	Initial fixation	Presentation time 150 ms / Upright presentation	Presentation time 50 ms / Inverted presentation
Fearful	Eyes	0.07	0.08
	Mouth	0.20	0.18
Happy	Eyes	0.09	0.08
	Mouth	0.12	0.18
Neutral	Eyes	0.09	0.08
	Mouth	0.21	0.18

Note. For the manipulation of the number of initial fixation positions, the average effect of the observed saccadic proportions of Experiment 1 was assumed.

Table S6. Results of the ANOVA on the proportion of saccades using the within-subject factors block, emotional expression, and initial fixation.

Effect	*df1*	*df2*	ε_{GG}	*F*	*p*	η_p^2
Emotion	2	86	.965	10.55	<.001***	.01
Initial fixation	1	43	-	4.92	.032*	.03
Block	1	43	-	2.02	.162	.00
Emotion × Initial fixation	2	86	.985	10.64	<.001***	.01
Emotion × Block	2	86	.962	0.23	.796	.00
Initial fixation × Block	1	43	-	16.33	<.001***	.01
Emotion × Initial fixation × Block	2	86	.968	5.26	.007**	.00

Note. *** $p < .001$, ** $p < .01$, * $p < .05$.

Table S7. Results of the ANOVA on unbiased hit rates using the within-subject factors block, emotional expression, and initial fixation.

Effect	*df1*	*df2*	ε_{GG}	*F*	*p*	η_p^2
Emotion	2	86	.553	3.06	.052	.07
Initial fixation	1	43	-	0.33	.569	.00
Block	1	43	-	0.24	.624	.00
Emotion × Initial fixation	2	86	.951	1.30	.279	.00
Emotion × Block	2	86	.883	0.26	.742	.00
Initial fixation × Block	1	43	-	0.98	.329	.00
Emotion × Initial fixation × Block	2	86	.810	0.72	.469	.00

Table S8. Results of the ANOVA on unbiased hit rates in block 1 using the within-subject factors emotional expression, and initial fixation.

Effect	*df1*	*df2*	ε_{GG}	*F*	*p*	η_p^2
Emotion	2	86	.793	4.56	.020*	.10
Initial fixation	1	43	-	0.69	.411	.02
Emotion × Initial fixation	2	86	.840	0.15	.820	.00

Note. * $p < .05$.

Table S9. Results of the ANOVA on unbiased hit rates in block 2 using the within-subject factors emotional expression, and initial fixation.

Effect	*df1*	*df2*	ε_{GG}	*F*	*p*	η_p^2
Emotion	2	84	.891	5.55	.007**	.12
Initial fixation	5	210	.894	0.76	.569	.02
Emotion × Initial fixation	10	420	.716	0.89	.520	.02

Note. ** $p < .01$.

Reaction Times

Similar to the analyses of unbiased hit rates, we first calculated a 2 × 3 × 2 ANOVA on response times of correct responses using the factors block, emotional expression and initial fixation location using comparable fixation locations of both blocks. This analysis only revealed significant main effects for emotional expression, $F(2, 84) = 18.43$, $\varepsilon = 1.00$, $p < .001$, $\eta_p^2 = .31$, and initial fixation, $F(1, 42) = 10.33$, $p = .003$, $\eta_p^2 = .20$. All other main and interaction effects were not statistically significant. Participants were faster when initially fixating on the mouth region and they were slower in correctly recognizing fearful facial expressions (see Figure S2).

Separate 2 × 3 and 3 × 6 ANOVAs for each block using the factors emotional expression and initial fixation location revealed significant main effects of emotional expression for block 1, $F(2, 86) = 14.17$, $\varepsilon = 1.00$, $p < .001$, $\eta_p^2 = .25$, and block 2, $F(2, 84) = 22.06$, $\varepsilon = .89$, $p < .001$, $\eta_p^2 = .04$, confirming generally slower responses to fearful facial expressions. For block 2, we additionally observed a significant main effect of initial fixation, $F(5, 210) = 3.80$, $\varepsilon = .88$, $p = .004$, $\eta_p^2 = .01$, and an interaction of both factors, $F(10, 420) = 1.82$, $\varepsilon = .69$, $p = .054$, $\eta_p^2 = .01$. These results indicate that participants were slower when initially fixation on the eye region, but this effect was most pronounced for neutral facial expressions.

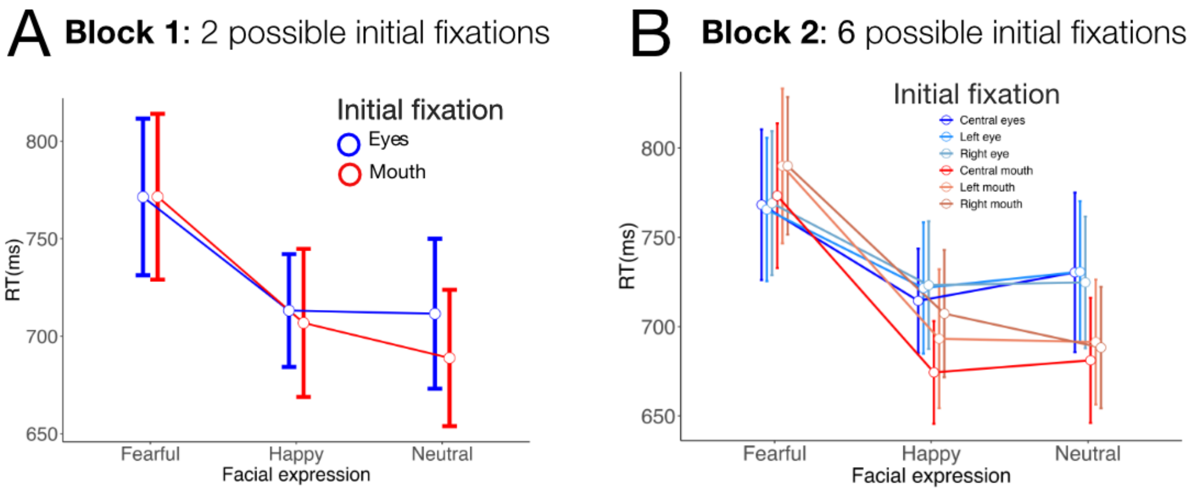

Figure S2: Comparison reaction time (RT) for initial fixation patterns across different facial expressions in two experimental blocks. **A.** Block 1 with two possible initial fixations, where participants initially fixated either on the eyes (blue) or the mouth (red**). B.** Block 2 with six possible initial fixations, including central, left, and right eye (blue shades) and central, left, and right mouth (red shades). Error bars represent 95%CI.

References

1. Lakens, D. & Caldwell, A. R. Simulation-Based Power Analysis for Factorial Analysis of Variance Designs. *Adv. Methods Pract. Psychol. Sci.* **4**, 2515245920951503 (2021).
2. Boll, S. & Gamer, M. 5-HTTLPR modulates the recognition accuracy and exploration of emotional facial expressions. *Front. Behav. Neurosci.* **8**, (2014).
